# Literary Notes

**Published:** 1896-08

**Authors:** 


### Literary Notes.

The *Scientific American* for July 25, 1896, is an edition *de luxe* commemorating the fiftieth anniversary of its foundation. Its first cover page is a symphony in art printing and illustration. Its coloring is soft and its design is appropriate and beautiful. The first reading page is devoted to reproductions of its issues for July 23 and August 28, 1846, while the remainder of its pages trace the improvement in methods of transit by land and sea, as well as in various industries, agriculture, astronomy, art, commerce, printing and other innumerable avenues of progress. An interesting illustration is one entitled Some distinguished inventors of the last half-century, in which we find medallions of Morse, Ericsson, Thompson, Howe, Bell, Harvey, Colt, Corliss, McCormick, Edison and Tesla, all grouped on a single plate. The projectors of the transatlantic cable is another historic picture that attracts attention and admiration. A full-page plate entitled Men of progress, contains the pictures of the principal inventors of the age and is full of attractiveness and furnishes food for much thought.

The *Scientific American* is to be congratulated in having maintained such an honorable place in scientific periodical literature for half a century, on the beautiful anniversary number that it publishes commemorative of its jubilee, and on the vast benefit it has bestowed on mankind.

The *Western Medical Review* issued its first number May 20, 1896. It is a well-printed double-column quarto and presents evidences of skilful management in its editorial department. The arrangement of its material is good and its selections for the most part are satisfactory; but we think it might have used its time and space to better advantage than to have printed Senn's abuse of the gynecologists in his Atlanta address. The first number of this magazine appears without advertisements, an unusual circumstance. We presume, however, that it will soon display these in goodly number and quality. We wish our new friend all the success that a work so well begun can and ought to command.

---

The New York Physicians' Mutual Aid Association has issued its twenty-seventh annual report, which shows a measure of prosperity that is truly remarkable. The total income from all sources during the year has been $21.293.88, while the entire running expenses have been only $1,465.66. There has been a net gain of 109 members during the year and the total membership January 3d was 1,333. It is difficult to understand how any physician in good repute should hold aloof from an association so economically managed and that provides such advantages.

---

The *Laryngoscope* is the name of a new journal that appeared in July, which is devoted entirely to the consideration of diseases of the nose, throat and ear. The *Laryngoscope* proposes to fill the niche between the strictly special and the general journals and is intended for physicians who confine themselves entirely to the treatment of the diseases mentioned or for those who pay special attention to those diseases while maintaining at the same time a general practice. It is published monthly at St. Louis and is edited by Drs. Frank M. Rumbold and M. A. Goldstein. The first number is a fine-looking medical magazine and appears as though it intended to fulfil its promises.

---

The *Students' Hand-Book* is the name of a little brochure issued by students in New York City as a pocket companion. It treats briefly regarding college and city and will be specially helpful to new students. The Students' Club is a homelike resort for students and is located at 129 Lexington avenue, New York City. It is open to all students, whether members or not, from 8 A. M. to 10 P. M. daily, where upper class men will be glad to give to new students a cordial welcome, together with such information about reliable boarding-houses and other matters of special interest to strangers.

---

The *Electrical Review* has just completed its twenty-eighth volume, which contains some of the best newspaper work ever done by a technical journal. In addition to giving thoroughly reliable news of the progress of electrical work in all its branches, the *Electrical Review* has secured in the past six months a large number of unusually valuable and exclusive articles on important subjects.

It printed the first official interview with Prof. Röntgen and the only interview with Prof. Salvioni, of the University of Perugia, Italy, who made some very interesting and remarkable discoveries on the Röntgen ray.

---

The *Archives of Clinical Skiagraphy* is a new candidate for professional favor. The object of this publication, says its editor, is to put on record in permanent form some of the most striking applications of the new photography to the needs of medicine and surgery. It is edited by Sydney Rowland, B. A., Camb., of London, and is published by the Rebman Publishing Co., Ltd., 11 Adam street Strand, London. We have received from the publishers the first two parts, May and June, 1896, which are beautiful specimens of medical magazine art. This publication is printed in quarto form, on heavy, highly finished book paper, with large-faced, handsome type, and the illustrations are artistic specimens of photomezzotypes, made by the London Stereoscopic Company. We have never seen the Röntgen X ray applied with such forcefulness and exactitude, nor developed in so much detail as in these pictures. No one interested in skiagraphy can afford to do without this magazine.

---

*Wilkinson's Medical Magazine* is the name and successor of the *Omaha Clinic*, which latter has been discontinued. If the new journal attains the excellence of its predecessor it may well be proud of itself.

---

The *New York Medical Journal* has lately appeared in a new dress that has given it a greatly improved appearance. This sterling medical weekly has justly attained the highest eminence in the field of medical journalism, and the new and improved dress referred to is an indication that the publishers propose to omit nothing that will serve to keep their journal always in the fore front.

---

It has been decided to erect in one of the squares of Paris a monument to Louis Pasteur. This tribute to the memory of Pasteur will be obtained through international effort. Dr. D. E. Salmon, chief of the bureau of animal industry, Washington, is chairman, and Dr. E. A. De Schweinitz, chief chemist, Biochemic laboratory, is the secretary of the American committee. The headquarters of the committee is at the Cosmos club, Washington, D. C. Subscriptions will be received by Dr. William Warren Potter, associate member of the committee, 284 Franklin street.

